# Psoriasis Flares in Patients With COVID-19 Infection or Vaccination: A Case Series

**DOI:** 10.7759/cureus.25987

**Published:** 2022-06-16

**Authors:** Hemali Shah, Ana C Busquets

**Affiliations:** 1 College of Medicine, Albany Medical College, Albany, USA; 2 Dermatology, Weirton Medical Center, Weirton, USA

**Keywords:** covid-19 vaccine, case series, biologics, psoriasis, covid-19

## Abstract

Much of the literature involving COVID-19 and chronic inflammatory dermatological conditions have focused on the safety of immunomodulatory therapy in the setting of this highly infectious virus. While general mortality associated with the infection and vaccine has been studied in depth, the effects of the virus and vaccine on inflammatory skin disease states have not been. It is well known that psoriasis can be triggered by stress, infection, certain medications, and, although not as common, vaccinations. Further, existing literature has briefly commented on psoriasis flares after COVID vaccination, but these have not touched on flares among their patients’ current therapy, nor flares after COVID infection. In this case report, we report five cases observed at our institution over the last year of either new-onset psoriasis or flares of previously well-controlled psoriasis shortly after infection with COVID-19 or COVID-19 vaccination, with no other identifiable triggers. These cases can serve to raise awareness of issues related to managing stubborn psoriatic flares and bring to the forefront conversations that are likely to arise with our patients regarding the risks and benefits of COVID vaccination and boosters. While the definitive etiology of the association between COVID and psoriasis remains unclear, it is important that the dermatologic community be aware when evaluating patients with new-onset or worsening psoriasis as we move forward in times of this COVID-19 era.

## Introduction

Much of the literature involving COVID-19 and chronic inflammatory dermatological conditions have focused on the safety of immunomodulatory therapy in the setting of this highly infectious virus. While general mortality associated with the infection and vaccine has been studied in depth, the effects of the virus and vaccine on inflammatory skin disease states have not been. Certainly, there are possible adverse effects of COVID vaccination with no long-term sequelae that are accepted as risks when compared to the severity of infection, but these have not been widely addressed in the literature [1,2].

It is well known that psoriasis can be triggered by stress, infection, certain medications, and, although not as common, vaccinations [1]. In this series, we report five cases observed at our institution over the last year of either new-onset psoriasis or flares of previously well-controlled psoriasis shortly after infection with COVID-19 or COVID-19 vaccination, with no other identifiable triggers. With this series, we hope to add to the limited existing literature by revealing that flares of psoriasis can occur in both untreated and chronically managed patients and as the result of both infection and vaccination.

## Case presentation

The first of our patients is a 22-year-old male who was well controlled on ustekinumab for four years. He was unvaccinated when he contracted a COVID-19 infection in December 2020. He developed a severe flare two weeks later that was not responsive to ustekinumab, risankizumab, or secukinumab, which is his current therapy. Further, he had an influenza infection in February 2021, which significantly worsened his flare. To date, his rash remains uncontrolled with plans to change therapy underway.

Next is a 70-year-old COVID-vaccinated male well controlled on apremilast for one year. He contracted a COVID-19 infection in August 2021 and flared one week later on his hands, left greater than right, which was similar to his initial presentation. His flare lasted four weeks and subsided without any additional therapeutic intervention. He had previously been vaccinated against COVID months earlier with no issues and received his booster shot one week prior to this office visit with no further flares.

The third patient is a 40-year-old COVID-vaccinated female with psoriasis refractory to secukinumab. She was then treated with adalimumab with improvement, but her course was then complicated by frequent urinary tract infections and worsening inverse psoriasis at the onset of the COVID pandemic. These isolated issues were managed and resolved with oral antibiotics and topical therapy. Because she was stable, she discontinued her immunobiological therapy due to personal concerns about tumor necrosis factor (TNF)-inhibitor therapy during the pandemic. She remained clear for seven months. Within two weeks of receiving her COVID booster vaccination, her psoriasis and psoriatic arthritis began to flare, with plaques re-appearing on her thighs and forearms, as well as below her breasts, leading to a body surface area (BSA) involvement similar to her refractory period presentations while on secukinumab. Plans to restart a TNF inhibitor are underway.

The fourth patient is a 51-year-old COVID-vaccinated female with longstanding, uncontrolled psoriasis for 20 years. She had been completely clear and stable on risankizumab for 2.5 years. She was infected with COVID despite being vaccinated months prior with no issues and noted her psoriasis began to flare several weeks after becoming infected. Her BSA involvement was limited enough to manage with mid- and high-potency topical corticosteroids.

The last patient is a 34-year-old COVID-vaccinated male who presented with new-onset psoriasis involving his scalp, ankles, knees, hips, and nails within one month of COVID infection. He had previously been vaccinated against COVID months prior to infection with no issues. His case is being successfully managed with topical clobetasol foam and calcipotriene cream. 

## Discussion

The hallmark of psoriasis is sustained inflammation led by a T-cell-driven autoimmune response with elevated levels of interleukin (IL)-23, IL-17, and TNF-a [1,3]. Known psoriasis triggers, such as infections, can indirectly affect the interplay of these mediators. Psoriasis has also been associated with higher levels of angiotensin-converting enzyme type 2 (ACE2) than the general population. COVID-19 spike protein has been noted to have a high affinity for ACE2 receptors. This could be a possible causal mechanism of reactivity in the association between psoriasis and COVID-19 infection and vaccination [4].

Vaccination is an uncommon trigger for psoriasis flares. However, there have been reports of psoriasis flares after vaccination for influenza, pneumococcal pneumonia, and yellow fever [1]. With the recent increase in mRNA vaccinations, McMahon et al. studied (N=414) cutaneous reactions after COVID-19 vaccination and found only two cases of psoriasis flares [5]. Wei and colleagues reported seven cases at their institution as well as investigated 79 cases reported in the Vaccine Adverse Events Reporting System. This report, however, did not specify whether patients were controlled on therapy prior to flare. Other studies have also shown that while patients have flared with vaccination, there have not been documented flares among patients on biologic therapy. Rather, this has been assessed and deemed to not be a correlation [6]. The patients observed at our institution, however, flared with infection or vaccination while on current biologic therapy in three out of five cases.

Psoriasis is also known to be triggered by infections. For example, the streptococcal infection has been associated with new-onset or flares of guttate psoriasis, which has been well reported in the literature [7]. A review article by Aram et al. found that flares of psoriasis were common after COVID infection, but these were largely attributed to the use of anti-malarial drugs or discontinuation of immunomodulatory therapy secondary to infection [8]. Our patients, in contrast, flared without the use of these medications or concurrent discontinuation of long-term therapy.

The pandemic has been a cause for irregular dermatological consultations and difficulty obtaining medications, and it has led to significant stress in the lives of many individuals, from social isolation to concerns about employment to the inability to seek medical care in a timely manner [9]. Psoriasis is often triggered by stress, including psychosocial stressors as well as physical stress on the body, which is important to note when addressing flares during the pandemic. However, all of our patients with these flares denied significant life or psychosocial stressors around the time preceding the onset of their psoriasis.

These cases are interesting for several reasons. First, the psoriasis flares noted occurred secondary to both COVID infection and vaccination. This suggests that one’s immune response to the virus is being replicated during vaccination; thus, these psoriatic manifestations are more certainly a result of the immune response rather than a direct viral assault. We, therefore, can likely continue to expect similar skin reactions whether a patient gets infected with COVID or receives the vaccination. Second, reactions happened in both stable, treated patients, as well as undiagnosed patients. It is possible that the hyper-inflammatory state induced by COVID-19 causes an upregulation of previously controlled cytokines, unmasking a genetic predisposition for psoriasis and that treatment with targeted anti-psoriatic systemic medication does not necessarily mitigate this risk.

## Conclusions

These cases can serve to raise awareness of issues related to managing stubborn psoriatic flares and bring to the forefront conversations that are likely to arise with our patients regarding the risks and benefits of COVID vaccination and boosters. Further, our cases show that these flares can occur in previously stable patients treated with immunobiologic therapies. While the definitive etiology of the association between COVID and psoriasis remains unclear, it is important that the dermatologic community be aware when evaluating patients with new-onset or worsening psoriasis as we move forward in times of this COVID-19 era, and we urge our colleagues to contribute to the literature surrounding this topic.
